# Association Between Time to First Mobilization and Recovery of Oral Intake Function in Patients with Pneumonia: A Two-Center Retrospective Cohort Study

**DOI:** 10.3390/life16040691

**Published:** 2026-04-20

**Authors:** Shinichi Watanabe, Takaaki Sakurai, Takahiro Kanaya, Takumi Iwasaki, Hyosuke Oshima, Tetsuya Furukawa, Tomohiro Yoshikawa, Seichi Nakahashi, Yasunari Morita

**Affiliations:** 1 Department of Rehabilitation, National Hospital Organization Nagoya Medical Center, 4-1-1 Sannomaru, Naka-ku, Nagoya 460-0001, Japan; 2 Department of Physical Therapy, Faculty of Rehabilitation, Gifu University of Health Science, 2-92 Higashiuzura, Gifu 500-8281, Japan; 3 Shibetsu Town Health and Welfare Center, Health Promotion Division, 1-2-6 North 1 West 5, Shibetsu-cho, Shibetsu-gun 086-1631, Japan; 4 Department of Rehabilitation Medicine, National Hospital Organization Hokkaido Medical Center, 5-7-1-1 Yamanote, Nishi-ku, Sapporo 063-0005, Japan; 5 Department of Emergency and Intensive Care Medicine, National Hospital Organization Nagoya Medical Center, 4-1-1 Sannomaru, Naka-ku, Nagoya 460-0001, Japan

**Keywords:** pneumonia, aspiration pneumonia, early mobilization, oral intake recovery, dysphagia, Functional Oral Intake Scale, frailty, rehabilitation

## Abstract

Delayed recovery of oral intake is common in hospitalized patients with pneumonia, particularly among older adults with reduced physical activity. Despite the recent emphasis on early mobilization, the relationship between the timing of first mobilization and recovery of oral intake function remains unclear. Thus, this retrospective cohort study investigated the association between time to first mobilization and recovery of oral intake in patients hospitalized with pneumonia. We analyzed 431 admitted patients with pneumonia, including aspiration pneumonia and coronavirus disease 2019 pneumonia, at two institutions. The Functional Oral Intake Scale ≥ 4 (partial oral intake recovery) was designated as the primary outcome. The main exposure was the number of days from admission to first mobilization. Multivariable Cox proportional hazards models were used after appropriate adjustments. The median time to first mobilization was 4 days (IQR: 2–14 days). Longer time to first mobilization was significantly associated with delayed recovery of oral intake (HR: 0.96, 95% CI: 0.94–0.98, *p* < 0.001). Thus, early mobilization may promote the recovery of oral intake in patients with pneumonia. These findings suggest that avoiding excessive delays in mobilization may support the recovery of oral intake and swallowing function in hospitalized patients with pneumonia.

## 1. Introduction

Pneumonia is a common disease among older adults and is frequently accompanied by functional decline and swallowing dysfunction in patients requiring hospitalization [1,2,3]. Particularly, reduced physical activity during hospitalization may accelerate disuse syndrome in these patients, resulting in delayed recovery of swallowing function and oral intake [4,5]. In recent years, similar concerns have been reported in patients with coronavirus disease 2019 (COVID-19) pneumonia, in whom disease severity and isolation management may lead to decreased physical activity and increased risk of dysphagia [6,7]. Consequently, comprehensive rehabilitation interventions for patients with pneumonia have received increasing attention.

Swallowing dysfunction is closely associated with recurrent aspiration pneumonia, malnutrition, and poor clinical outcomes, including prolonged hospitalization [8,9]. Therefore, early recovery of oral intake is an important clinical goal in the management of patients with pneumonia. In addition to swallowing rehabilitation and nutritional management, early mobilization and promotion of physical activity have recently been suggested to contribute to improvements in overall physical function and swallowing recovery [10,11,12]. Early mobilization may attenuate muscle weakness and sarcopenia, and improve respiratory function and consciousness levels, thereby indirectly facilitating recovery of swallowing function.

Physical therapists play a key role in the mobilization and exercise therapy of patients with pneumonia during the acute phase of hospitalization. Although the importance of rehabilitation in patients with pneumonia has been widely recognized, the relationship between the timing of mobilization and the recovery process of oral intake function remains unclear. Previous studies have examined the association between early mobilization or time to first mobilization and outcomes such as physical function or length of hospital stay [13,14]. However, most of these studies have focused on functional outcomes or prognosis, and studies that specifically evaluate swallowing function or recovery of oral intake as primary outcomes remain limited [15].

Although early mobilization has been widely studied in patients with pneumonia and in acute care settings, most previous studies have primarily focused on general functional outcomes such as physical function, activities of daily living, or length of hospital stay [13,14]. In contrast, the recovery of swallowing function and oral intake has received comparatively less attention, despite its critical importance for nutritional status, prevention of recurrent aspiration, and overall prognosis. Furthermore, while mobilization and physical activity are considered important components of rehabilitation, their relationship with swallowing recovery has not been sufficiently investigated. In particular, it remains unclear whether the timing of mobilization is independently associated with recovery of oral intake in patients with pneumonia. Therefore, the present study specifically focuses on recovery of functional oral intake (FOIS ≥ 4) as a clinically meaningful outcome and employs restricted cubic spline analysis to explore potential nonlinear associations between time to first mobilization and oral intake recovery. This study aimed to investigate the association between the time to first mobilization and recovery of functional oral intake in patients hospitalized with pneumonia.

## 2. Materials and Methods

### 2.1. Study Design and Participants

This retrospective observational study was conducted at two hospitals. Patients admitted with pneumonia between January and December 2022 were retrospectively screened for eligibility. Pneumonia cases included aspiration pneumonia and coronavirus disease 2019 (COVID-19) pneumonia. The attending physicians at each institution diagnosed the type of pneumonia based on clinical symptoms, imaging findings, and laboratory results. Patients with COVID-19 pneumonia were included because they were managed under similar clinical pathways and rehabilitation protocols as other types of pneumonia during the study period, reflecting real-world clinical practice.

Among patients admitted during the study period, those who received physical therapy during hospitalization and had available data on the timing of their first mobilization and oral intake status were included in the analysis. Patients who were unable to take oral intake prior to admission, those with severe neurological diseases that precluded the evaluation of swallowing function at admission, terminally ill patients, those requiring intubation after admission, and those with gastrointestinal diseases requiring prohibition of oral intake for more than three days after admission were excluded.

Clinical data were retrospectively extracted from electronic medical records at each participating hospital. Both institutions used similar electronic medical record systems and data structures, allowing for consistent data collection and variable definitions across sites. Data extraction was performed by designated speech–language–hearing therapists at each institution. When necessary, the extracted data were reviewed and cross-checked within the research team to ensure accuracy and consistency. The study period was limited to a fixed 12-month duration to ensure data completeness and consistency in clinical practice across participating institutions. This study used clinical data obtained during routine medical care. No additional interventions or treatment modifications were performed for research purposes.

### 2.2. Outcome Measures

#### 2.2.1. Primary Outcome

The primary outcome was recovery of functional oral intake, assessed using the Functional Oral Intake Scale (FOIS). The FOIS is a 7-point scale (1–7) that evaluates oral intake. In this study, functional oral intake recovery was defined as FOIS ≥ 4, indicating partial oral intake [16].

The outcome was defined as the number of days from hospital admission to the first achievement of FOIS ≥ 4. Patients who did not reach FOIS ≥ 4 during hospitalization were censored at discharge. Clinical data were retrospectively extracted from electronic medical records at each participating hospital. Data extraction was performed by designated speech–language–hearing therapists at each institution. When necessary, the extracted data were reviewed and cross-checked within the research team to ensure accuracy and consistency.

#### 2.2.2. Primary Exposure

The primary exposure variable was time to first mobilization, defined as the first activity at the level of sitting on the edge of the bed or higher from the day of admission. Accordingly, the number of days from admission to first mobilization was calculated [17,18].

#### 2.2.3. Covariates

The following variables were included as covariates: age, sex, body mass index (BMI), pneumonia type (non-aspiration pneumonia, aspiration pneumonia, or COVID-19 pneumonia), pre-admission Clinical Frailty Scale (CFS) [19], FOIS at admission, serum albumin level at admission, time to first mobilization, time to initiation of oral intake, and length of hospital stay. Serum albumin was measured at admission and expressed in g/dL, and was treated as a continuous variable in the analysis without categorization. These variables were selected based on previous studies and clinical relevance [20,21].

### 2.3. Statistical Analyses

Continuous variables are presented as medians with interquartile ranges (IQRs), and categorical variables are presented as counts and percentages. The association between time to first mobilization and time to recovery of functional oral intake (FOIS ≥ 4) was examined using Cox proportional hazards models. Time to first mobilization was treated as a continuous variable. Multivariable models were adjusted for age, sex, pre-admission Clinical Frailty Scale (CFS), pneumonia type, serum albumin level at admission (g/dL), time to initiation of physical therapy, and hospital site. Results are presented as hazard ratios (HRs) with 95% confidence intervals (95% CIs), where an HR greater than 1 indicates a higher probability of earlier achievement of FOIS ≥ 4.

To evaluate potential nonlinear associations, restricted cubic spline functions were incorporated into the multivariable Cox proportional hazards model. Four knots were placed at predefined quantiles of the distribution of time to first mobilization, in accordance with commonly recommended statistical practices. The median value (4 days) was selected as the reference point to facilitate clinically interpretable comparisons. Model-predicted hazard ratios were plotted across the range of the exposure variable.

All statistical analyses were performed using JMP software (version 13.0; SAS Institute Inc., Cary, NC, USA). Statistical significance was defined as a two-sided *p*-value of < 0.05.

### 2.4. Ethical Considerations

This study was conducted in accordance with the Declaration of Helsinki and approved by the Ethics Committee of Nagoya Medical Center (Institutional Review Board approval number: 2022061; approved on 9 May 2023). As this study used anonymized clinical data obtained during routine medical care, the requirement for informed consent was waived. The patients were provided with an opportunity to opt out of the study.

## 3. Results

### 3.1. Participant Characteristics

**Figure 1:**
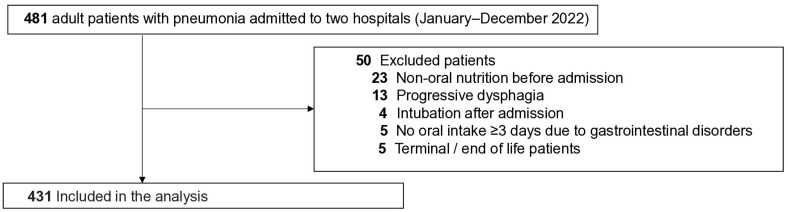
Flow chart of the study.

In total, 431 patients were included in the analysis (Figure 1). The median age was 84 years (IQR: 76–89 years), and approximately 60% of the participants were male. The study population included patients with non-aspiration pneumonia, aspiration pneumonia, and COVID-19 pneumonia.

The median pre-admission CFS score was 6 (IQR: 5–7), indicating that many patients had moderate-to-severe frailty. The median time to first mobilization was 4 days (IQR: 2–14 days). The median time to the initiation of oral intake was 3 days (IQR: 1–15 days), and the median time to the initiation of physical therapy was 2 days (IQR: 1–7 days). The median serum albumin level at admission was 3.1 g/dL (IQR: 2.6–3.5 g/dL) (Table 1).

### 3.2. Association Between Time to First Mobilization and Recovery of Oral Intake

The association between time to first mobilization and achievement of functional oral intake (FOIS ≥ 4) was examined using a multivariable Cox proportional hazards model (Table 2). A longer time to first mobilization was independently associated with delayed recovery of oral intake. Specifically, each 1-day delay in first mobilization was associated with a significantly lower probability of achieving FOIS ≥ 4 (HR: 0.96, 95% CI: 0.94–0.98, *p* < 0.001).

Among the covariates, the pre-admission CFS was also significantly associated with the outcome. Each one-point increase in CFS was associated with a lower probability of achieving FOIS ≥ 4. Male sex tended to be associated with delayed recovery of oral intake compared to female sex. In contrast, age, serum albumin level at admission, time to initiation of oral intake, and time to initiation of physical therapy were not independently associated with the outcome.

### 3.3. Nonlinear Association Between Time to First Mobilization and Recovery of Oral Intake

A restricted cubic spline Cox proportional hazards model was used to evaluate the nonlinear association between time to first mobilization and recovery of functional oral intake (FOIS ≥ 4).

**Figure 2:**
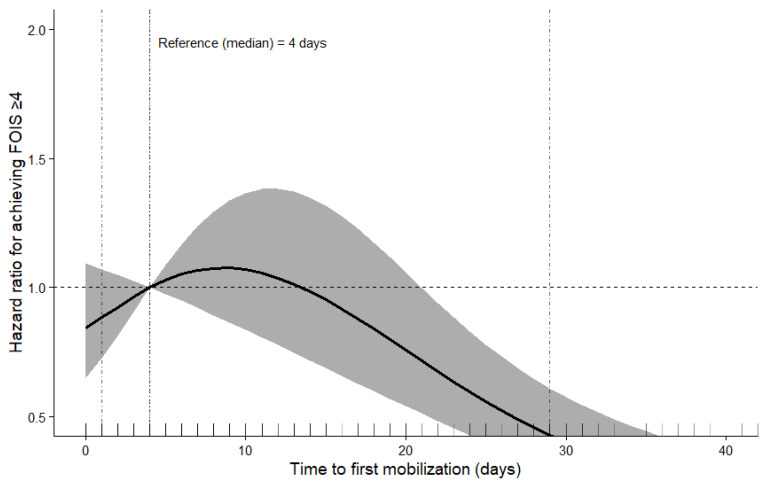
Restricted cubic spline analysis of the association between time to first mobilization and recovery of functional oral intake (FOIS ≥ 4). The solid line represents the adjusted hazard ratio (HR), and the shaded area indicates the 95% confidence interval. The median time to first mobilization (4 days) was used as the reference value. HRs below 1 indicate a lower probability of achieving functional oral intake recovery compared to the reference. The model was adjusted for age, sex, Clinical Frailty Scale, pneumonia type, serum albumin level, time to initiation of physical therapy, and hospital site.

Time to first mobilization was significantly associated with achievement of FOIS ≥ 4 (overall Wald test, *p* = 0.002), and the association showed a nonlinear pattern (Figure 2). Compared with the reference value (median: 4 days), the hazard of achieving FOIS ≥ 4 gradually decreased as time to first mobilization increased, with a consistent decline observed particularly after approximately 10 days. In contrast, mobilization before the reference value did not result in a clear additional increase in HR.

## 4. Discussion

In this study, we investigated the association between the time to first mobilization and recovery of functional oral intake in patients hospitalized with pneumonia. The results demonstrated that a longer time to first mobilization was independently associated with delayed achievement of functional oral intake (FOIS ≥ 4). This association remained significant even after adjusting for potential confounders, including age, sex, pre-admission frailty, nutritional status, time to initiation of physical therapy, and hospital site.

An important finding of this study is that the timing of mobilization was associated with recovery of swallowing and oral intake, which are functional outcomes related to nutrition and rehabilitation. Previous studies have mainly focused on the effects of early mobilization or rehabilitation interventions on outcomes, such as physical function or length of hospital stay [22,23]. In contrast, the recovery of swallowing function and oral intake has often been considered within the domains of nutritional management and speech–language therapy, and the relationship between mobilization or exercise therapy provided by physical therapists and swallowing recovery has not been sufficiently investigated [24].

Patients with pneumonia represent a unique clinical population characterized by advanced age, frailty, systemic inflammation, and reduced physical activity [5]. These factors are closely associated with both dysphagia and delayed recovery of oral intake [2]. In particular, aspiration risk and respiratory dysfunction are highly prevalent in this population, distinguishing them from other disease groups. Unlike other populations, recovery of oral intake in patients with pneumonia is influenced not only by direct swallowing rehabilitation but also by systemic factors such as physical activity, mobilization, and overall functional status [4]. Therefore, the timing of mobilization may play a particularly important role in facilitating recovery of oral intake in this population. The present findings suggest that the timing of mobilization, which is largely coordinated by physical therapists, may also be related to the recovery processes in the swallowing and nutritional domains.

Several mechanisms may explain the association between delayed mobilization and delayed recovery of oral intake. First, delayed mobilization may lead to prolonged physical inactivity, which can accelerate the progression of sarcopenia and disuse syndrome [5]. The muscles involved in swallowing are closely related to overall muscle strength and postural control, and reduced physical activity may negatively affect swallowing function. Second, mobilization that enables patients to achieve a sitting or standing posture may improve consciousness levels and respiratory function, thereby facilitating safe initiation and continuation of oral intake [25]. Collectively, these mechanisms suggest that delayed mobilization may adversely affect the recovery of swallowing function and oral intake.

Another notable finding of this study is the nonlinear relationship observed between time to first mobilization and recovery of oral intake. Using restricted cubic spline analysis, we found that the hazard of achieving FOIS ≥ 4 gradually decreased as the time to first mobilization increased, particularly after approximately 10 days. In contrast, mobilization earlier than the reference value did not show a clear additional benefit in achieving FOIS ≥ 4. These findings suggest that the timing of mobilization, rather than its presence or absence, may be important for the recovery of oral intake. In particular, an excessive delay in mobilization may be associated with an unfavorable recovery of oral intake.

The absence of a clear additional benefit of very early mobilization may be explained by several factors. The recovery of oral intake in the early phase of hospitalization is strongly influenced by factors such as systemic inflammation, level of consciousness, respiratory status, and underlying disease severity [26,27]. Therefore, the timing of mobilization alone may not fully determine the rate of recovery of oral intake. In addition, the outcome used in this study (FOIS ≥ 4) represents a functional threshold indicating partial oral intake, and a ceiling effect may have limited the ability to detect additional benefits of very early mobilization.

These findings suggest that clinical management should not necessarily focus solely on achieving the earliest possible mobilization, but rather on avoiding excessive delays in initiating mobilization while considering patient safety and clinical stability. Therefore, timely initiation of mobilization may represent an important strategy to support the recovery of swallowing function and oral intake in patients with pneumonia.

Another strength of this study is that the analysis was conducted using multicenter data, including patients with aspiration pneumonia and COVID-19 pneumonia. These patient populations are often associated with advanced age, disease severity, and activity restrictions owing to infection control measures. The finding that the timing of mobilization is associated with oral intake recovery, even in heterogeneous populations, provides clinically meaningful insights for rehabilitation practice.

These findings suggest that clinical management should not necessarily focus solely on achieving the earliest possible mobilization, but rather on avoiding excessive delays in initiating mobilization while considering patient safety and clinical stability. Therefore, timely initiation of mobilization may represent an important strategy to support the recovery of swallowing function and oral intake in patients with pneumonia. Another strength of this study is that the analysis was conducted using multicenter data, including patients with aspiration pneumonia and COVID-19 pneumonia. These patient populations are often associated with advanced age, disease severity, and activity restrictions owing to infection control measures. The finding that the timing of mobilization is associated with oral intake recovery, even in heterogeneous populations, provides clinically meaningful insights for rehabilitation practice.

This study has several limitations. First, this was an observational study, and the causal relationship between delayed mobilization and recovery of oral intake could not be definitively established. Although we adjusted for several clinically relevant covariates, residual confounding cannot be excluded. In particular, factors such as disease severity, clinical decision-making regarding the timing of mobilization, and patients’ overall condition may have influenced both the exposure and the outcome. Therefore, the observed associations should be interpreted with caution. Second, mobilization was defined as sitting on the edge of the bed or higher; however, the amount of physical activity or exercise intensity after mobilization was not evaluated. Third, the frequency of swallowing assessments and details of the rehabilitation interventions may have differed across institutions. Future prospective or interventional studies are needed to clarify whether earlier mobilization directly contributes to improved recovery of swallowing function and oral intake.

## 5. Conclusions

In this study, the time to first mobilization was independently associated with the recovery of functional oral intake in patients hospitalized with pneumonia. In particular, delayed initiation of mobilization beyond a certain period (>10 days) was associated with slower recovery of functional oral intake (FOIS ≥ 4). These findings suggest that the timing of mobilization, in which physical therapists play a key role, may influence not only physical function, but also the recovery process of swallowing and nutritional status. Therefore, appropriate mobilization during the early phase of hospitalization may be important for promoting the recovery of oral intake in patients with pneumonia.

## Figures and Tables

**Table 1:** Baseline Characteristics of the Study Participants.

Variables	Overall (*n* = 431)
Age, years	84 (76–89)
Male, *n* (%)	247 (57.3)
BMI, kg/m^2^	19.7 (17.2–22.5)
Pneumonia type, *n* (%)	
Non-aspiration pneumonia	87 (20)
Aspiration pneumonia	161 (37)
COVID-19 pneumonia	168 (39)
Others	15 (4)
Pre-admission Clinical Frailty Scale	6 (5–7)
FOIS at admission	2 (1–5)
Serum albumin at admission, g/dL	3.1 (2.6–3.5)
Time to first mobilization, days	4 (2–14)
Time to initiation of physical therapy, days	2 (1–7)
Length of hospital stay, days	20 (13–34)

Data are presented as median (interquartile range) or number (%). First mobilization was defined as the first activity at the level of sitting on the edge of the bed or higher. BMI, body mass index; COVID-19, coronavirus disease 2019; FOIS, Functional Oral Intake Scale.

**Table 2:** Association between Time to First Mobilization and Recovery of Functional Oral Intake.

Model 1. Linear Model (Effect Per 1-Day Delay)			
Variables	HR	95% CI	*p*-Value
Time to first mobilization (per 1-day delay)	0.96	0.94–0.98	<0.001
Age (per 1-year increase)	0.99	0.98–1.01	0.465
Male sex	0.75	0.60–0.95	0.016
Pneumonia type (non-aspiration pneumonia)	0.74	0.54–1.01	0.062
Pneumonia type (aspiration pneumonia)	1.20	0.65–2.07	0.536
Pneumonia type (COVID-19 pneumonia)	1.15	0.51–1.47	0.437
Clinical Frailty Scale (per 1-point increase)	0.81	0.76–0.87	<0.001
Serum albumin at admission (g/dL, per 1 g/dL increase)	0.99	0.83–1.19	0.974
Time to initiation of physical therapy (per 1-day delay)	1.01	0.98–1.03	0.992
**Model 2. Nonlinear Model (Restricted Cubic Spline)**			
**Variables**	**Overall ** ***p*** **-Value**		**Nonlinear ** ***p*** **-Value**
Time to first mobilization	0.002 (Wald χ^2^ = 14.67)		<0.001

Model 1 shows the adjusted association between time to first mobilization and recovery of functional oral intake. Hazard ratios were estimated using a multivariable Cox proportional hazards model adjusted for age, sex, Clinical Frailty Scale, pneumonia type, serum albumin at admission, time to initiation of physical therapy, and hospital site. Model 2 evaluated potential nonlinear associations using restricted cubic spline functions with four knots placed at the quantiles of time to first mobilization. The median value (4 days) was used as the reference. HR, hazard ratio; CI, confidence interval; FOIS, Functional Oral Intake Scale; CFS, Clinical Frailty Scale; COVID-19, coronavirus disease 2019. An HR greater than 1 indicates a higher probability of earlier recovery of functional oral intake.

## Data Availability

The data supporting the findings of this study are available from the corresponding author upon reasonable request. The data are not publicly available due to privacy and ethical restrictions.

## Abbreviations

The following abbreviations are used in this manuscript:

BMI	Body Mass Index
CFS	Clinical Frailty Scale
CI	Confidence Interval
COVID-19	Coronavirus Disease 2019
FOIS	Functional Oral Intake Scale
HR	Hazard Ratio
IQR	Interquartile Range
RCS	Restricted Cubic Spline

## References

[1] Marik P.E., Kaplan D. (2003). Aspiration pneumonia and dysphagia in the elderly. Chest.

[2] Altman K.W., Yu G.P., Schaefer S.D. (2010). Consequence of dysphagia in the hospitalized patient: Impact on prognosis and hospital resources. Arch. Otolaryngol. Head Neck Surg..

[3] Langmore S.E., Skarupski K.A., Park P.S., Fries B.E. (2002). Predictors of aspiration pneumonia in nursing home residents. Dysphagia.

[4] Yagi M., Yasunaga H., Matsui H., Fushimi K., Fujimoto M., Koyama T., Fujitani J. (2016). Effect of early rehabilitation on activities of daily living in patients with aspiration pneumonia. Geriatr. Gerontol. Int..

[5] Momosaki R. (2017). Rehabilitative management for aspiration pneumonia in elderly patients. J. Gen. Fam. Med..

[6] Dziewas R., Warnecke T., Zürcher P., Schefold J.C. (2020). Dysphagia in COVID-19 -multilevel damage to the swallowing network?. Eur. J. Neurol..

[7] See E.J., Bellomo R. (2021). How I prescribe continuous renal replacement therapy. Crit. Care.

[8] Maeda K., Akagi J. (2016). Sarcopenia is an independent risk factor of dysphagia in hospitalized older people. Geriatr. Gerontol. Int..

[9] Suzuki K., Miyamoto M., Hirata K. (2017). Sleep disorders in the elderly: Diagnosis and management. J. Gen. Fam. Med..

[10] Schweickert W.D., Pohlman M.C., Pohlman A.S., Nigos C., Pawlik A.J., Esbrook C.L., Spears L., Miller M., Franczyk M., Deprizio D. (2009). Early physical and occupational therapy in mechanically ventilated, critically ill patients: A randomised controlled trial. Lancet.

[11] Hodgson C.L., Stiller K., Needham D.M., Tipping C.J., Harrold M., Baldwin C.E., Bradley S., Berney S., Caruana L.R., Elliott D. (2014). Expert consensus and recommendations on safety criteria for active mobilization of mechanically ventilated critically ill adults. Crit. Care.

[12] Adler J., Malone D. (2012). Early mobilization in the intensive care unit: A systematic review. Cardiopulm. Phys. Ther. J..

[13] Needham D.M., Korupolu R., Zanni J.M., Pradhan P., Colantuoni E., Palmer J.B., Brower R.G., Fan E. (2010). Early physical medicine and rehabilitation for patients with acute respiratory failure: A quality improvement project. Arch. Phys. Med. Rehabil..

[14] Morris P.E., Goad A., Thompson C., Taylor K., Harry B., Passmore L., Ross A., Anderson L., Baker S., Sanchez M. (2008). Early intensive care unit mobility therapy in the treatment of acute respiratory failure. Crit. Care Med..

[15] Crary M.A., Mann G.D., Groher M.E. (2005). Initial psychometric assessment of a functional oral intake scale for dysphagia in stroke patients. Arch. Phys. Med. Rehabil..

[16] Hamzic S., Braun T., Juenemann M., Butz M., Voswinckel R., Belly M., Vogelbusch O., Weber S., Khilan H., Keps M. (2021). Validation of the German Version of Functional Oral Intake Scale (FOIS-G) for Flexible Endoscopic Evaluation of Swallowing (FEES). Dysphagia.

[17] Ad Hoc Committee for Early Rehabilitation, Japanese Society of Intensive Care Medicine (2017). Evidence-based expert consensus for early rehabilitation in the intensive care unit. Jpn. J. Intensive Care Med..

[18] Hodgson C.L., Bailey M., Bellomo R., Brickell K., Broadley T., Buhr H., Gabbe B.J., Gould D.W., Harrold M., TEAM Study Investigators and the ANZICS Clinical Trials Group (2022). Early active mobilization during mechanical ventilation in the ICU. N. Engl. J. Med..

[19] Rockwood K., Song X., MacKnight C., Bergman H., Hogan D.B., McDowell I., Mitnitski A. (2005). A global clinical measure of fitness and frailty in elderly people. CMAJ.

[20] Fujishima I., Fujiu-Kurachi M., Arai H., Hyodo M., Kagaya H., Maeda K., Mori T., Nishioka S., Oshima F., Ogawa S. (2019). Sarcopenia and dysphagia: Position paper by four professional organizations. Geriatr. Gerontol. Int..

[21] Needham D.M., Davidson J., Cohen H., Hopkins R.O., Weinert C., Wunsch H., Zawistowski C., Bemis-Dougherty A., Berney S.C., Bienvenu O.J. (2012). Improving long-term outcomes after discharge from intensive care unit: Report from a stakeholders’ conference. Crit. Care Med..

[22] Nydahl P., Sricharoenchai T., Chandra S., Kundt F.S., Huang M., Fischill M., Needham D.M. (2017). Safety of patient mobilization and rehabilitation in the intensive care unit: Systematic review with meta-analysis. Ann. Am. Thorac. Soc..

[23] Castro-Avila A.C., Serón P., Fan E., Gaete M., Mickan S. (2015). Effect of early rehabilitation during intensive care unit stay on functional status: Systematic review and meta-analysis. PLoS ONE.

[24] Nakamura T., Kurosaki S. (2020). Effects of early dysphagia rehabilitation by speech-language-hearing therapists on patients with severe aspiration pneumonia. Prog. Rehabil. Med..

[25] Park B.H., Seo J.H., Ko M.H., Park S.H. (2013). Effect of 45° reclining sitting posture on swallowing in patients with dysphagia. Yonsei Med. J..

[26] Kortebein P., Ferrando A., Lombeida J., Wolfe R., Evans W.J. (2007). Effect of 10 days of bed rest on skeletal muscle in healthy older adults. JAMA.

[27] Brown C.J., Friedkin R.J., Inouye S.K. (2004). Prevalence and outcomes of low mobility in hospitalized older patients. J. Am. Geriatr. Soc..

